# Embolic Stroke of Uncommon Causes—A Case Series Report from an Acute Stroke Unit

**DOI:** 10.3390/life16060930

**Published:** 2026-06-01

**Authors:** Mihaiela Lungu, Anamaria Ionescu, Luminita Lăcrămioara Apostol, Andrei Lucian Zaharia, Elena Niculet, Violeta Diana Oprea

**Affiliations:** 1 Neurological Department, “St. Ap. Andrew” County Emergency Clinical Hospital in Galati, 800578 Galati, Romania; micalungu@gmail.com; 2 Clinical Medical Department, Faculty of Medicine and Pharmacy, “Dunarea de Jos” University of Galati, 800008 Galati, Romania; 3 Research Centre in the Medical-Pharmaceutical Field, Faculty of Medicine and Pharmacy, “Dunarea de Jos” University of Galati, 800008 Galati, Romania; 4 Faculty of Medicine, University Ovidius Constanta, 900470 Constanta, Romania; iuliusana@gmail.com; 5 “St. Ap. Andrew” County Emergency Clinical Hospital in Galati, 800578 Galati, Romania; lum.apostol@gmail.com; 6 Medical Department, Faculty of Medicine and Pharmacy, “Dunărea de Jos” University of Galati, 800008 Galati, Romania; 7 Department of Morphological and Functional Sciences, Faculty of Medicine and Pharmacy, “Dunarea de Jos” University of Galați, 800008 Galati, Romania; helena_badiu@yahoo.com; 8 Pathology Department, “St. Ap. Andrew” County Emergency Clinical Hospital in Galati, 800578 Galati, Romania; 9 Geriatric Department, Sjællands Universitetshospital Nykøbing Falster, 4800 Nykobing Falster, Denmark

**Keywords:** acute cerebral ischemia, acute stroke, embolic stroke of undetermined source, ESUS, cardioembolic stroke, vascular injury stroke, uncommon stroke mechanisms

## Abstract

Background: Beyond the well-known etiologies of ischemic stroke associated with recognized vascular risk factors, the literature describes a spectrum of uncommon causes with low prevalence but significant diagnostic and therapeutic implications. Materials and methods: A case series report of seven rare etiopathogenic mechanisms (in patients aged 25–85) who presented within a 3-year interval (2022–2025) to our stroke unit at the Neurological Department—“St. Ap. Andrew” County Emergency Clinical Hospital in Galati, Romania. Results: The seven uncommon embolic stroke cases included three patients with infectious cerebral ischemia (hydatid cyst with double simultaneous embolism, mycotic aneurysm of the carotid artery, syphilis-associated stroke), three cases of rare cardioembolic strokes (patients with Lambl excrescences, cor triatriatum, left ventricular non-compaction), as well as one patient with multiple etiological associations triggering acute stroke. Conclusions: Rare etiologies of ischemic stroke should be actively considered when classical vascular risk factors are absent, particularly in young patients. Comprehensive diagnostic evaluation is mandatory, especially in cases of embolic stroke of undetermined source (ESUS). Such cases represent significant diagnostic and therapeutic challenges and require a multidisciplinary approach to optimize patient outcomes.

## 1. Introduction

Uncommon etiologies of ischemic stroke comprise a heterogeneous group of mechanisms that extend beyond classical causes such as large-artery atherosclerosis, small-vessel disease, and common cardioembolic sources. Although rare, these conditions are particularly relevant in younger patients and in cases of cryptogenic stroke, where conventional vascular risk factors are missing.

Among these ischemic mechanisms, rare cardiovascular sources represent one of the most frequently encountered categories [1]. Structural cardiac abnormalities may act as embolic sources even without overt heart disease. For example, Patent Foramen Ovale has been associated with paradoxical embolism, allowing venous thrombi to enter the arterial circulation. Other rare cardiac causes include tumors such as Atrial Myxoma and valvular lesions like Nonbacterial Thrombotic Endocarditis [2,3].

Rare genetic and hereditary disorders are also an important cause, particularly in early-onset stroke. Examples include cerebral autosomal dominant arteriopathy with subcortical infarcts and leukoencephalopathy (CADASIL), mitochondrial diseases such as mitochondrial encephalomyopathy with lactic acidosis and stroke-like episodes (MELAS) syndrome, and metabolic disorders like Fabry Disease, which affect vascular integrity and cellular metabolism [2,4].

Additional mechanisms include hypercoagulable states, such as antiphospholipid syndrome, and hematologic diseases like Sickle Cell Disease. Non-atherosclerotic vasculopathies, including Moyamoya Disease and Fibromuscular Dysplasia, may also predispose to ischemia [4].

Finally, infectious mechanisms are recognized as rare causes of stroke, often mediated by vascular inflammation. Examples include Varicella-Zoster Virus Vasculopathy, Neurosyphilis, and Tuberculous Meningitis [1,2,5].

Generally, we might suspect an uncommon cause of a stroke in young age with unexplained stroke, recurrent strokes or strokes in unusual locations, presence of severe headaches or other neurological signs like visuospatial deficit or known family history of stroke or clotting disorders. Awareness of these uncommon stroke etiologies is essential because it enables accurate diagnosis, targeted and personalized management, and effective prevention of recurrence, especially in young or atypical patients.

The aim of this article is to highlight several uncommon causes of embolic stroke where the identification of the etiopathogenic mechanism was relevant for patients’ clinical and therapeutic management, assessment of prognosis and the mitigation of the risk of recurrence. The retrospective selection of these cases, while not exhaustive, may help the readership to consider relevant differential diagnosis which could actually add a new perspective for the management algorithms and medium to long-term prognosis of stroke patients.

## 2. Materials and Methods

This case series report presents seven rare—and thus challenging—diagnoses of uncommon etiologies of stroke, from the Acute Stroke Unit/UAVCA Center within the Neurological Department—“St. Ap. Andrew” County Emergency Clinical Hospital in Galati, Romania (within the last 3 years 2022–2025, from a total of 4460 acute stroke cases). Some of these rare causes are just only reported in the available literature in a few articles or present some unexpected associations, generating stroke through multiple etiopathogenic mechanisms.

Specific diagnostic investigations were performed for each case. Relevant imagistic or histopathology figures were included in each case report.

For each etiology, significant data from a review of the available literature in relevant databases (PubMed and Embase) was also presented. Search criteria included key words for each etiopathogeny, applied for a timeframe covering the last relevant publications starting with 1960.

## 3. Results: Case Reports and Discussions

Among the rare etiologies that may cause cerebral ischemia, we included in this case series report three etiopathogenic categories—Table 1:

### 3.1. Systemic and Central Nervous System Infections (CNS)

Systemic and CNS infections may precipitate ischemic stroke through inflammatory activation, hypercoagulability, endothelial dysfunction, or direct vascular involvement. The precise mechanisms remain incompletely elucidated, and outcomes are often unfavorable. Infectious agents capable of inducing cerebral ischemia include bacterial, parasitic, viral, and fungal pathogens, which may act either as predisposing or triggering factors [6].

#### 3.1.1. Case 1.1: Rupture of a Hydatid Cyst with Simultaneous 2-Sites Embolism

Human cystic echinococcosis (hydatidosis) is an increasingly prevalent zoonosis, partly due to population migration. Epidemiologic data for Romania, considered a highly endemic country for hydatidosis (cited annual incidence between 3.3 and 5.6 cases in 100,000 inhabitants) are varying from one region to another, depending on how much the population is engaged in farming and agricultural activities [6]. Hydatid cysts may be located in multiple organs, and clinical manifestations depend on cyst size, age, and anatomical location [7,8,9].

Cyst rupture may result in embolization of hydatid membrane fragments, mass effect, anaphylaxis, or sudden death. Simultaneous embolization to cerebral arteries (causing ischemic stroke) and peripheral arteries is exceptionally rare, particularly when associated with anaphylactic shock. The usual source is a cyst located in the left atrium, pericardium, or mediastinum [8,9].

We report on the case of a 25-year-old male patient presenting with symptoms relevant for acute stroke, associating right upper limb ischemia. In our case, acute right upper limb ischemia, raising the suspicion of an embolism in the right brachial artery, with sudden onset within the day preceding the presentation to the hospital, while the patient also complained of severe headache, resulted in diagnosing a hydatid cyst rupture with simultaneous embolism in a cerebral artery, in the brachial artery and with associated anaphylactic manifestations. Cerebral imaging confirmed a posterior cerebral artery territory infarction. The patient was a farmer with no other medical history except a posttraumatic left brachio-brachial bypass performed in 2015.

CT-scanning of the thorax showed, over the left atrium, a cyst with a diameter of 69 mm, while at the level of the apex and continuing postero-superior, in direct contact with the pericardium, three agglutinated formations—a cystic complex of 80/43/96 mm—were detected.

Acute cerebral ischemia was accompanied by anaphylactic manifestations and acute brachial artery ischemia requiring emergency surgical revascularization. Histopathological examination of the arterial thrombi visualized anhistous hydatid membrane fragments, confirming the embolic mechanism.

**Figure 1:**
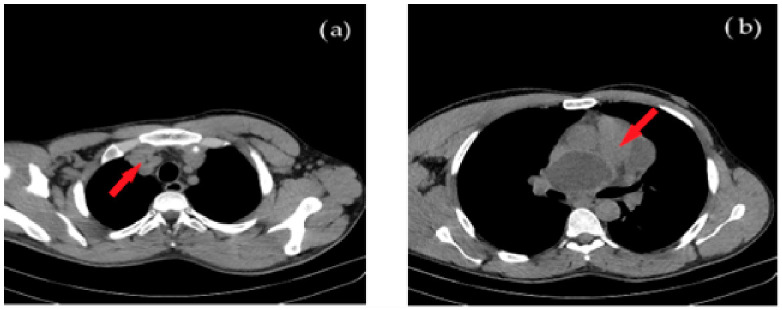
(**a**,**b**): Contrast and native CT of thorax and abdomen show multiple (**a**) mediastinal and (**b**) pericardial hydatic cysts.

**Figure 2:**
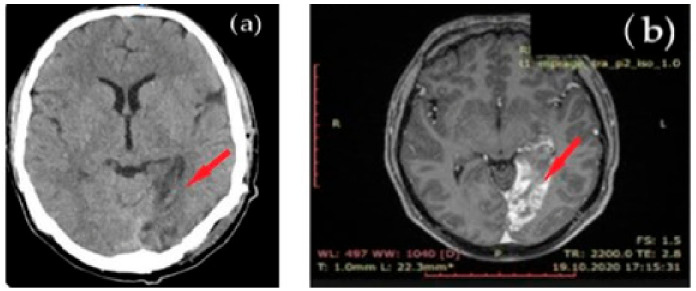
(**a**,**b**): Cerebral imaging. (**a**) Native brain CT aspect: left occipital ischemic hypodense lesion; (**b**) brain MRI aspect: left occipital ischemia.

Simultaneous embolisms—cerebral (generating stroke) and in the arteries of the limbs, (with the appearance of acute ischemia phenomena), are rarely cited in the literature. Even rarer are reports with associated phenomena of anaphilaxis at the time of cyst rupture. The starting point in these cases are, predominantly, cysts located in the left atrium, pericardium or mediastinum—which was also our patient’s situation: Figure 1a,b:

Stroke suggestive symptomatology was confirmed by brain imaging—Figure 2a,b.

**Figure 3:**
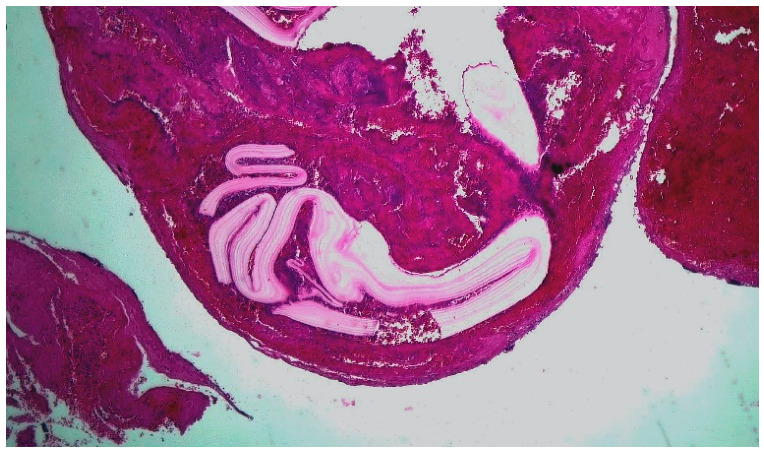
Histopathological examination of the hydatid cyst membrane collected from the brachial artery: fibrin clot enclosing fragments of the anhistous membrane and blood clot fragment (HE × 40 stain).

Acute cerebral ischemia was associated, in this case, with anaphylactic shock and with acute brachial artery ischemia, which required emergency intervention. The histology samples from the brachial artery confirm the embolism with fragments of hydatid cyst membrane—Figure 3:

Post-surgery, the patient had a favorable evolution, with the restoration of the blood flow in the right brachial artery and the remission of the hemianopsia.

The patient continued as per standard therapeutic echinococcosis protocols with albendazole and was recommended regular imagistic monitoring of therapy efficacy and surveillance of the existing pericardial and mediastinal lesions.

Discussion:

Cardiac hydatid cysts account for approximately 0.5–2% of echinococcosis cases, most frequently involving the right ventricle. Peripheral arterial embolization is extremely rare, with only few cases reported. The concomitant onset of neurological deficits, peripheral ischemia, and allergic manifestations strongly supports cyst rupture as the causal event [10].

Cardiac hydatid cyst location in the right ventricle is usually subendocardial, the rupture of these cysts being more frequent than in other intracardiac locations. Embolisms in the peripheral arteries are extremely rare; in the literature only embolisms in the arterial axis of the lower limbs are reported. Acaturk et al. presented a case of ischemic stroke due to rupture of a cardiac hydatid cyst [10], similar cases being reported by Singh et al. 2003 [11] and Maffeis et al. 2000 [12]. However, a retrospective study from the Barcelona Stroke Study considers that up to 6% of all cerebral ischemia is caused by the rupture of the hydatid cyst.

Peripheral arterial embolism in the brachial artery is a rare clinical presentation, the literature citing only several cases involving the femoral or popliteal arteries [13,14]. Additionally, we identified very few reports of stroke due to embolism after a ruptured hydatid cyst [10,11].

#### 3.1.2. Case 1.2: Mycotic Aneurysm of the Carotid Artery

Mycotic aneurysms of the extracranial carotid artery are rare but carry a high mortality risk. Early clinical manifestations are often nonspecific and may include septic embolization caused by organisms such as *Staphylococcus aureus*, *Streptococcus* spp., *Haemophilus*, *Salmonella*, or *Proteus* [15,16].

**Figure 4:**
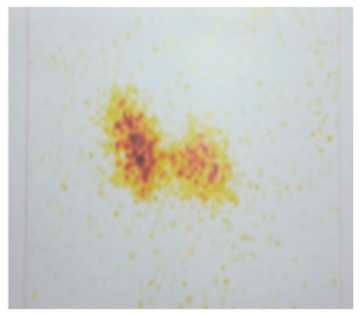
Thyroid scintigraphy with hypo-uptake in the left thyroid lobe, slight hypo-uptake in the right lobe, without suspect scintigraphy nodules.

**Figure 5:**
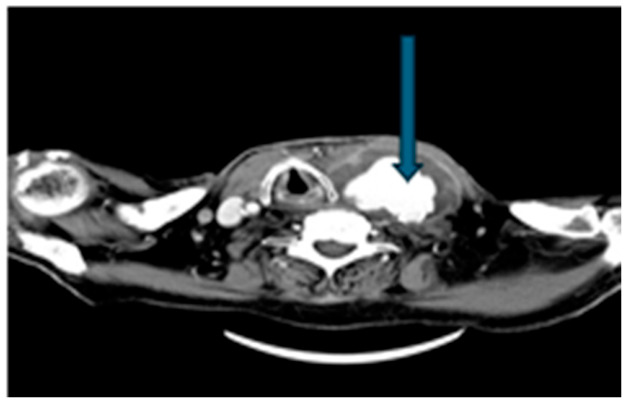
Left latero-cervical mass capturing the contrast substance with deviated esophagus and trachea.

**Figure 6:**
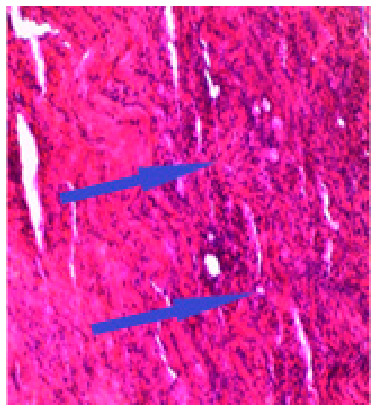
Fragment of carotid wall, presenting rich inflammatory infiltrate, missing the normal histological architecture (surgical excision piece, staining HE × 40).

We present the case of a 77-year-old patient with myelodysplastic syndrome and chronic corticosteroid therapy, who acutely installed a left carotid ischemia with right hemiparesis, and after 12 h chills, fever and left lateral-cervical inflammatory nodules. At the time of admission, due to the evidence of severe anemia and thrombocytopenia, the patient required blood transfusion. The tonsillar phlegmon was ruled out and, for differential diagnosis with strumitis, thyroid scintigraphy was performed—Figure 4:

The CT scanning of the cervical region showed a voluminous left latero-cervical mass, capturing the contrast substance, surrounding the left common carotid artery—Figure 5:

The multidisciplinary assessment suspected a left carotid mycotic aneurysm; surgery was performed identifying a dissection of the carotid wall at the fungal aneurysm site, with carotid involvement at the bifurcation and rich inflammatory tissue—Figure 6.

These lesions occur more frequently in immunocompromised patients, intravenous drug users, in children after severe upper respiratory infections, and patients with endocarditis or prior carotid interventions. In our unit, this patient with myelodysplastic syndrome and chronic corticosteroid therapy developed acute carotid territory ischemia, followed by fever and cervical inflammatory swelling. Imaging revealed a ruptured mycotic aneurysm at the carotid bifurcation. Despite urgent surgical intervention, the patient died within 24 h. Blood cultures identified methicillin-resistant *Staphylococcus aureus* bacteriemia.

Discussion:

Ruptured fungal aneurysm of the extracranial carotid artery wall, complicated with cerebral ischemia in the afferent carotid axis is rare and has a significant risk of death [15,16,17]. The initial clinical manifestations may be nonspecific, with septic embolization with the etiological agent occurring as *Staphylococcus aureus*, *Pneumococcus*, *Haemophilus*, *Salmonella*, *Proteus* or with unidentified germs. It is more common in children after streptococcal angina with pharyngeal abscess, jugular vein thrombophlebitis, after carotid stenting, in immuno-compromised patients, drug users or patients with endocarditis [17,18,19,20,21,22]. It can also involve carotid fascia, with contralateral transmission.

Bacterial colonization of atherosclerotic plaques promotes biofilm formation, plaque destabilization, and aneurism dissection. Early diagnosis and aggressive multidisciplinary management are essential to improving survival [15,19,21].

#### 3.1.3. Case 1.3: Syphilis-Associated Stroke

The incidence of syphilis has increased worldwide over the last 20–25 years, this increase being accompanied by a resurgence of neurosyphilis. Symptomatic neurosyphilis is more likely to occur with longer duration of illness but can occur at any stage of the infection.

Between 2010 and 2023, ten patients with neurosyphilis were diagnosed in our neurology clinic presenting with ischemic stroke—Table 2.

We present the case of the oldest patient—an 85 year-old man, also the most recent syphilis-associated stroke in our clinic, who had no cardiovascular risk factors. Suddenly, 2 months before the stroke episode, the patient needed a coronary stent—probably due to luetic vasculitis.

At the moment of stroke diagnosis, the patient presented thrombocythemia and positive TPHA in CSF, with affected arterial territory in the left sylvian artery (confirmed by angio-MRI). The patient’s family informed that the patient had had behavioral changes debuting 4 months prior, accompanied by nocturnal psychomotor agitation and slowly progressing mnesic impairment. The patient received high-dose intravenous penicillin and corticotherapy. At the 1-month follow-up visit, the patient presented severe right hemiplegia and global aphasia, and the patient was then lost to follow-up.

Discussion:

The number of syphilis cases have been increasing in Europe over the last 20 years, frequently associated with HIV infection [23,24]. Cerebral infarction occurs in about 15% of patients with neurosyphilis, typically 5–12 years after the first infection [23,24]. Neurosyphilis should be considered in patients—particularly young individuals without traditional vascular risk factors—who present with stroke involving the middle cerebral artery territory. Diagnosis requires confirmation of Treponema pallidum Hemagglutination Assay (TPHA) positivity in cerebrospinal fluid (CSF). Key points regarding the experience in our center:

- Residual deficits are common, especially in older patients or those with recurrent infarcts.
- Even with early antibiotic therapy, neurologic sequelae such as hemiparesis, aphasia, and cognitive impairment may persist.

### 3.2. Rare Cardioembolic Causes

Accounting for roughly 20% of ischemic strokes, those with cardioembolic mechanisms are often severe, with high recurrence rates and a tendency for sudden onset often involving multiple brain territories. Common causes include atrial fibrillation (AF), mechanical valves, and recent heart attacks [25,26].

Cardioembolic strokes of unusual cause are rare embolic events (<1% of cases) originating from atypical cardiac sources, including cardiac tumors (e.g., atrial myxoma), papillary fibroelastoma, mitral annular calcification, paradoxical embolism via a patent foramen ovale (PFO), or thrombus on a Eustachian valve. These, alongside rare genetic disorders and hypercoagulable states, require extensive investigation, often involving transesophageal echocardiography (TEE) to identify the source in order to allow the specific management [25,26].

#### 3.2.1. Case 2.1: Stroke Associated with Lambl Excrescences (LE)

Lambl excrescences (LE) are filamentous valvular structures arising at valve closure lines, most commonly affecting the aortic valve. They possess significant embolic potential and are best detected using transesophageal echocardiography. Although frequently asymptomatic, LE may cause embolic events. Anticoagulation is recommended after a first embolic episode, while surgical management should be considered following recurrence [25,26].

**Figure 7:**
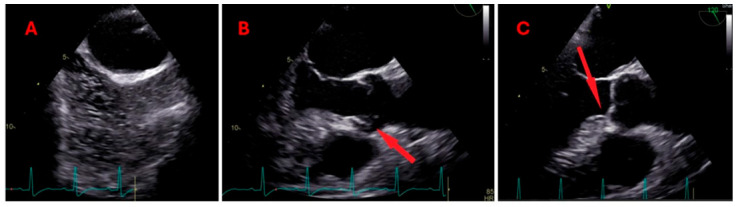
(**A**): Transthoracic echocardiography—bicav section: no detectable shunt at IV injection of bubbled glucose serum. (**B**,**C**): Transesophageal cardiac ultrasound (TEE)—Lambl excrescences at the level of the aortic cusps ((**B**)—parasternal long-axis view, (**C**) short axis level of the aortic valve).

Our 67-year-old male patient presented with right hemiparesis, mixed transcortical aphasia, without known cardiovascular risk factors. Transesophageal cardiac ultrasound (TEE) in our patient with acute ischemic stroke detected: aortic valve—supple tricuspid; filamentous formation attached to the non-coronary cube—LE, mitral valve with minimal regurgitation A2P2, without detectable shunts (including upon injecting bubbled glucose serum)—Figure 7A–C:

The clinical evolution was of a regressive stroke type, with full recovery within a few days. The patient did not present cardiovascular risk factors, and continued the dual antiplatelet therapy with a recommendation of yearly TTE follow-up.

Discussion:

Currently, it is considered that LE increases in frequency with age and is more frequently identified due to patients’ access to investigation methods [26,27]. Most patients with LE are asymptomatic, but those located on the aortic valve become most commonly embolic [25,26]. LE can cause embolic stroke and requires transesophageal echocardiographic investigations. The first embolic event benefits from anticoagulant treatment, while in the second, the surgical sanction must be considered and performed according to the particularities of the case [26]. A case reported for a left middle cerebral artery infarct within the Wernicke area, followed by a second stroke which occurred ten months later involving left lenticulo-striate arteries area, in a woman with initial TEE showing a mitral valve lesion suggesting a thrombus, was demonstrated by pathologic examination as having LE as the cause of cerebral embolization [27].

#### 3.2.2. Case 2.2: Cerebral Ischemia in Cor Triatriatum with Associated Structural Abnormalities

The three-atria heart (cor triatriatum) is a rare congenital cardiac anomaly, characterized by the presence of an abnormal intra-atrial membrane dividing the atrial cavity, with involvement of the cor atriatum sinister type—the most common, or cor atriatum dexter, more rarely [28,29]. Clinical presentation depends on membrane morphology and associated cardiac defects.

In our clinic, a 55-year-old female patient with known autoimmune thyroiditis and celiac disease, a 38 pack-year smoking history, presenting with right hemiparesis, ASPECTS 10, with NIHSS 6 points at initiation and 4 points at 24 h, underwent thrombolysis. After thrombolysis, hypodense lesions were installed posteriorly in the caudate nucleus and the left external capsule.

We present this patient case with incomplete cor triatriatum sinister, ostium primum atrial septal defect, left atrial appendage thrombosis, mitral valve abnormalities, and extensive aortic atheromatosis who presented with embolic ischemic stroke. The etiology was multifactorial, conferring a substantial embolic risk. Long-term anticoagulation was initiated, considering that generally surgical intervention is reserved for selected cases (not suitable for our case). The patient is continuing follow-up in a cardiology clinic.

**Figure 8:**
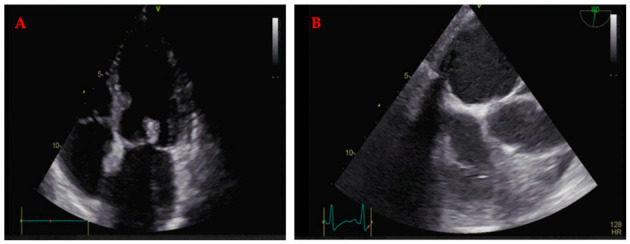
(**A**,**B**) Transthoracic ultrasound—four-chamber section, rearranged mitral valve, dilated left atrium (**A**). Transesophageal ultrasound—filamentous formation at the left atrium level, attached to the posterior face—aspect compatible with cor triatriatum sinister (**B**).

**Figure 9:**
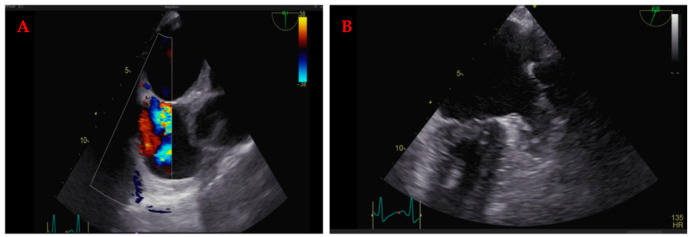
(**A**,**B**) Transesophageal ultrasound. (**A**) Biconcave section—small hole visible in the “primum” portion of the interatrial septum, connected to the membrane in the left atrium. (**B**) Transesophageal ultrasound-recent thrombosis at the level of the LAA.

Transesophageal ultrasound detected the left atrium and the left auricle with echoes of stasis, recent thrombosis of the left atrial appendage (LAA), a filamentous formation inside the left atrium, an aspect compatible with the triatriatum sinister heart. Right-left shunt is evident at the level of the interatrial septal defect, both as a color Doppler image and as a contrast bubble from the right atrium to the left atrium—Figure 8 and Figure 9.

Discussion:

The incidence for cor triatriatum sinistrum is less than 0.1% of heart diseases diagnosed in childhood [28,29]. In adults, the condition of cor triatriatum becomes symptomatic only if fibrosis or calcification of the accessory membrane orifice occurs, manifestations including dyspnea, orthopnea, and hemoptysis. The anomaly can be highlighted due to ischemic events or heart rhythm disorders (atrial fibrillation)—in our case embolic stroke [28].

#### 3.2.3. Case 2.3: Left Ventricular Non-Compaction with Right Parietal Stroke

Left ventricular non-compaction (LVNC) is a rare cardiomyopathy characterized by excessive myocardial trabeculation resulting from arrested embryonic myocardial compaction. There are numerous excessive trabeculations and intertrabecular recessus, which communicate with the left ventricular cavity [30]. The process of cardiomyocyte compaction is physiologically finalized during embryogenesis, while in LVNC disturbances generate the phenotype of increased left ventricular trabecular tissue [31,32,33]. Clinical manifestations include heart failure, arrhythmia, and systemic thromboembolism [31,32,33].

**Figure 10:**
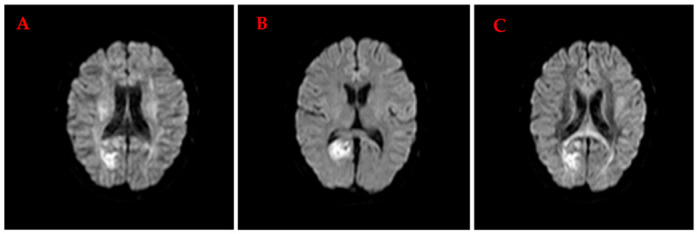
(**A**–**C**) Cerebral MRI T2-Weighted (T2W): Area of right parasagittal T2 hypersignal with recent ischemia (**A**). Cerebral MRI T2-Weighted (T2W): Right parasagittal parietal ischemic lesion **B**,**C**).

**Figure 11:**
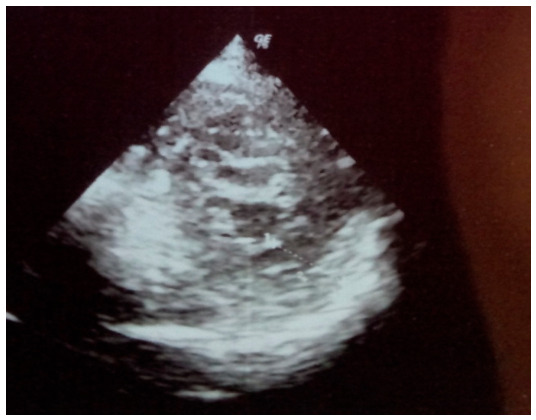
Cardiac ultrasound appearance of LVNC, with trabeculates.

**Figure 12:**
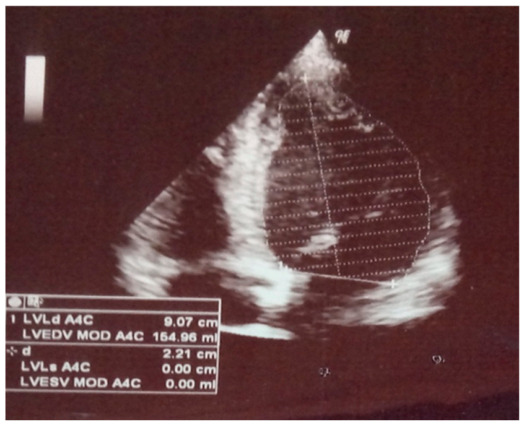
Cardiac ultrasound appearance of LVNC (left ventricle area).

A 40-year-old male patient, with no significant medical history, presented in our clinic with acute ischemic stroke, and the cardiac ultrasound revealed typical features of LVNC. Anticoagulation therapy was initiated, with favorable neurological recovery. Cerebral MRI detected an area with T2 hypersignal and FLAIR, with liquid diffusion restrictions on the diffusion sequences, right parietal parasagittal location, suggesting recent ischemic lesion, with normal venous sinuses—Figure 10A–C:

The transthoracic echocardiogram detected, at the level of the left ventricle, apical numerous trabeculae through which the penetration of the color Doppler signal is observed: compact zone/compact + non-compact zone, section SAX = 9/21 + 9 = 0.3. Conclusions: the left ventricle is not dilated, in relation to the body surface, but excessively apical trabeculates, with preserved systolic function, EF = 67%; mild diastolic dysfunction, delayed relaxation type, no significant hemodynamic valvopathies, no pulmonary hypertension estimated by surrogate parameters, free pericardium—Figure 11 and Figure 12.

Considering the cardiac ultrasound examination, as well as the exclusion of other etiologies for cerebral ischemia following all investigations, the parietal ischemic lesion was diagnosed as a cerebral embolic event determined by LVNC.

Antiplatelet treatment was initiated, later opting for oral anticoagulation. The evolution was favorable, with improvement of visual field changes and sensory syndrome.

Discussion:

Such embolic events are of rare occurrence in LNVC; the literature review shows that the condition is associated with a variable prognosis worsened by an increased risk of cardioembolic events. Anticoagulation is recommended even in the absence of documented atrial fibrillation. Some studies associate the disease with high mortality due to heart failure and sudden cardiac death [34]. The prognosis depends on the stage of the disease diagnosis, the severity of heart failure and the improvements due to treatment.

The incidence of LNVC cases is very low, and of these cases, only 25% are cited as presenting, in evolution, an episode of stroke through the cardioembolic mechanism [30,33,34].

The American Heart Association (AHA) has classified LVNC as a primary genetic cardiomyopathy while The European Society of Cardiology (ESC) considers it as an unclassified cardiomyopathy, because LVNC may be a morphological manifestation of other severe distinct cardiomyopathies [30].

Studies in the genetics of LVNC have strongly suggested that the disease has an inheritance pattern (18% to 50% of cases are familial) [6]. Several studies suggest that LVNC is a heterogeneous genetic disease, with familial and sporadic form, with pathogenic mutations in genes encoding cytoskeletal, mitochondrial, sarcomeric and Z-line proteins. Various modes of autosomal dominant, recessive, x-linked or mitochondrial transmission have been described [30,35]. In sporadic cases, which are common, de novo mutations have been detected [30]. There is the absence of specific genotype–phenotype association in LVNC; the mutation of the same gene can cause both LVNC and dilated or hypertrophic cardiomyopathy, which makes genetic testing of limited utility. Three genes related to LVNC have been identified: alpha-dystrobrevin (DTNA), cypher/ZASP (a Z-line component gene in both skeletal and cardiac muscle), and TAZ (gene with unknown dysfunction, also involved in x-linked dilated cardiomyopathy) [30,35].

The clinical triad formed by symptoms of heart failure, arrhythmia and cardioembolic events represents the manifestation in patients with diastolic dysfunction of the left ventricle [30]. Various types of arrhythmias can occur, from atrial fibrillation (7–26%), to persistent ventricular tachycardia [36]. In a long-term follow-up of 34 adults with isolated LVNC by Oechslin et al., there were 5 cardiac deaths, 16 heart failure hospitalization, 10 ventricular arrhythmias and 5 thromboembolic events [34]. Transitory ischemic attacks or stroke in 25% of all patients with LVNC have been reported in two series [31,34]. Because of the incidence of 29% atrial fibrillation in LVNC patients, systemic anticoagulation is recommended.

### 3.3. Stroke Due to Multiple Etiological Associations (Patient Case 3)

Some cases of ischemic strokes result from the coexistence of several predisposing factors. We report the case of a patient with bilateral paramedian thalamic infarction consistent with the occlusion of the artery of Percheron (a rare anatomical variant of thalamic vascular supply), associated with patent foramen ovale (PFO), antiphospholipid syndrome with high-risk thrombophilia (having a spontaneous abortion in her personal medical history), and dyslipidemia. Percutaneous PFO closure and tailored antithrombotic therapy were used in the management of this case.

**Figure 13:**
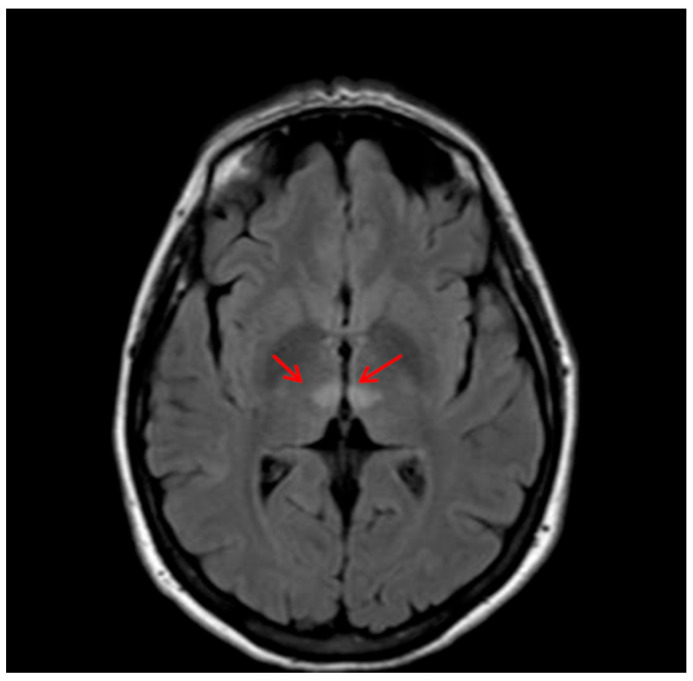
Cerebral MRI with the appearance of bilateral thalamic ischemia—Percheron artery. T2 sequence.

**Figure 14:**
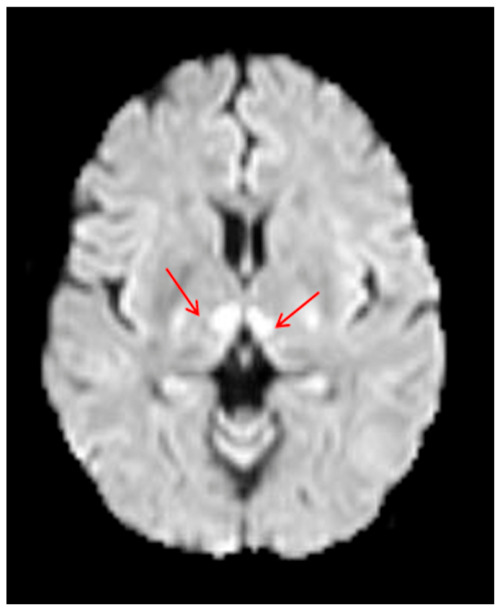
Cerebral MRI with the appearance of bilateral thalamic ischemia—Percheron artery. FLAIR sequence.

While hospitalized, this 46-year-old female patient presented sudden onset vertigo, syncope, somnolence and, subsequently, coma GCS-6, later with the resumption of consciousness, but with marked ataxia. The patient’s cerebral MRI presents at bilateral thalamic level, two lesions in T2 hypersignal, FLAIR and DWI, with T1 hyposignal and discrete ADC hyposignal, well defined, without adjacent edema and without mass effect on neighboring structures, with diameters between 9 and 11 mm—Figure 13 and Figure 14:

The right vertebral artery was hypoplastic, and the cervical segment of the left internal carotid artery (ICA) had a 16% stenosis.

**Figure 15:**
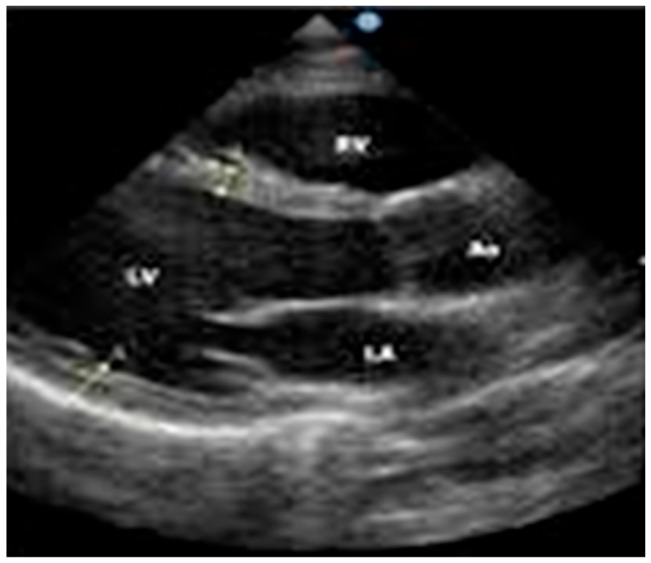
Cardiac transesophageal ultrasound—Foramen ovale slope with intermittent, right-to-left blood shunting.

After confirming a bilateral thalamic ischemic stroke by MRI angiography, a cardiac ultrasound was performed, showing grade 1 mitral regurgitation, grade 1 tricuspid regurgitation, non-dilated ESR with normal global and segmental systolic function, ejection fraction (EF) 65%, and small PFO tunnel—Figure 15:

The transesophageal ultrasound (Figure 15) showed that, when the contrast agent was administered intravenously in the right forearm, the right atrium became opaque and the passage of a small number of bubbles in the left atrium was observed, in the second cardiac cycle after the Valsalva maneuver.

An antiphospholipid syndrome was detected with positive beta 2glycoprotein Ig A antibodies at 3 successive determinations, but also high-risk thrombophilia, with homozygous positive MTHFR A 1298C mutation and hyperhomocysteinemia, the patient also having dyslipidemia. Percutaneous closure of the PFO was used with the COCOON 15–18 mm device and ASA 100 mg/day was administered with clopidogrel in combination for 3 months, as well as statin.

Discussion:

Percheron artery occlusion is a rare cause of ischemic stroke characterized by bilateral paramedian thalamic infarctions. It usually presents with changes in mental status, hyper-drowsiness and eye movement disorders. AOP occlusion may be suspected in a patient with a low conscious level, ophthalmological signs and cardio-embolic risk factors [36].

In this case, there is a complex association of possible etiologies of a stroke in the vertebrobasilar system, with a probable embolic mechanism and with risk factors such as thrombophilia and antiphospholipid syndrome, plus the anatomical variant of the Percheron artery. In such situations, it is difficult to determine which could be the main etiopathogenic mechanism of the stroke, so all the factors involved should be managed appropriately, for an improved prognosis and secondary prevention for future events.

## 4. Discussion

Beyond atrial fibrillation and other recognized major risk factors, embolic stroke may also have uncommon causes, presented in the recent literature while diagnostic tools became more performant and broadly available in the last period.

Still, the majority of rare causes for stroke have cardiac & vascular sources: paradoxical embolism—when clot travels from vein to artery via a shunt (Patent Foramen Ovale—PFO, Atrial Septal Defect—ASD), cardiac tumors (especially left atrial myxoma), endocarditis, ventricular issues (thrombus after myocardial infarction or due to a cardiomyopathy -like doxorubicin-induced disorder), aortic arch atheromas or fibromuscular dysplasia (FMD)—leading to arterial narrowing or tears, often in carotids. Other hematologic disorders resulting in blood clotting at cerebral level include hypercoagulable states—both inherited (Factor V Leiden, Protein C/S/Antithrombin deficiency) or acquired (antiphospholipid syndrome, polycythemia, thrombocythemia), and sickle cell disease (SCD) [36,37].

Uncommon stroke etiologies (often classified under “other determined causes” in frameworks like the TOAST system = Trial of Org 10172 in Acute Stroke Treatment classification system) require an expansive diagnostic approach because their pathways intersect with multiple medical specialties [38,39]. Shared pathways and key clinical indicators generally include systemic inflammatory/infectious pathways, hematological/coagulation pathways or vascular/cardiovascular structural pathways.

Terminology used for Embolic Stroke of Undetermined Source (ESUS) describes non-lacunar ischemic strokes presenting imaging features that are suggestive of embolism, occurring in patients without major cardioembolic risk. ESUS is a subset, but does not equal cryptogenic stroke, requiring therefore a complete diagnostic workup to exclude identifiable etiologies [36]. In order to qualify as an ESUS, the stroke has to check some precise criteria, like No major blockage in the arteries, No high-risk heart sources, and No other specific cause—i.e., the stroke cannot be attributed to other rare causes like drug misuse, arterial dissection, or migraines.

Patients presenting with ESUS may have a considerable risk for stroke recurrence as well as for other serious cardiovascular events, which emphasize even more the importance of optimizing preventive strategies to mitigate this high risk [37]. The widespread use of modern diagnostic imaging techniques and availability of several medical and interventional strategies that could potentially reduce stroke risk in ESUS-associated pathologies (like supracardiac atherosclerosis, PFO, left atrial/ventricular disease, cardiac valvular disease, infectious diseases and cancers with emboligenic potential) identify ESUS as an important priority in stroke research of the near coming future. “Cryptogenic stroke” includes ESUS but also patients with multiple etiologies, such as our final case report presented or, for example, patients with both atrial fibrillation and atherosclerotic carotid stenosis ipsilateral to the infarct, and this also includes patients with incomplete diagnostic workup [37,38].

Some of these presented case reports could have remained cases of ESUS/cryptogenic strokes (i.e., ischemic strokes of unknown origin) if some symptoms or extensive investigations would have been overlooked or non-available or simply performed at a different moment of clinical evolution.

As patients with few traditional risk factors may be classified as cryptogenic, clinical, radiographic, and risk factor profiles vary. Thorough etiological investigations and appropriate classification are needed to best categorize these patients. In the absence of conventional risk factors and in patients with ESUS, ancillary testing such as use of TEE, C-MRI is required for cardiac imaging, while extensive, prolonged cardiac monitoring should be considered in all patients assessed as ESUS. Stroke patients with associated systemic manifestations suggesting infectious or inflammatory conditions may benefit from CSF examination, and screening for hypercoagulable state in younger patients with recurrent thrombosis or family history may be appropriate [37,38,39].

Because misclassification or delayed etiologic diagnosis is directly correlated with elevated rates of recurrent ischemic events, refractory cases necessitate a comprehensive, longitudinal diagnostic review. Utilizing modernized investigative frameworks and advanced phenotyping systems, as outlined in current consensus statements, is paramount to refining secondary prevention strategies and optimizing patient outcomes.

## 5. Conclusions

Before diagnosing a stroke as cryptogenic or ESUS, extensive medical investigations should be performed in order to identify a possible rare cause of emboly whenever modifiable factors are not present, or there are no conditions in the patient’s personal or hereditary history that would justify an etiopathogenic mechanism for the occurrence of cerebral ischemia. These causes should be especially considered in young people in whom other etiologies are excluded. Complex investigations are necessary to establish such etiologies, especially in the case of ESUS. The identification of the etiopathogenic mechanism can contribute to identifying the correct treatment and evaluation of the prognosis but also mitigating the risk of future recurrence.

When conventional diagnostic protocols fail to elucidate the precise etiology of an ischemic stroke, the diagnostic paradigm must evolve from basic syndromic classification to an iterative, highly targeted phenotypic investigation. Ultimately, reducing the burden of recurrent stroke hinges on resolving diagnostic ambiguity; therefore, challenging presentations demand the rigorous application of updated diagnostic algorithms to successfully transitions patients from an empirical ‘cryptogenic’ classification to a mechanism-specific therapeutic regimen.

## Figures and Tables

**Table 1:** Overview of case series and main diagnostic tools used for each.

Etiopathogenic Mechanism	Case Reported	Demographic	Diagnostic Protocol
Section 3.1 Systemic and Central Nervous System Infections (CNS)	Section 3.1.1 Rupture of a hydatid cyst with simultaneous 2-sites embolism	25/M/farmer	CT-scanning (cerebral and thorax/abdomen), histopathological examination
	Section 3.1.2 Mycotic aneurysm of the carotid artery	77/M/retired taxi driver	CT-scanning (cerebral and cervical/thorax region), histopathology, thyroid scintigraphy
	Section 3.1.3 Syphilis-associated stroke	85/M/retired	Cerebral MRI, CSF analysis (lumbar puncture), serological testing
Section 3.2 Rare cardioembolic causes	Section 3.2.1 Stroke associated with Lambl excrescences (LE)	67/M/mechanic	Cerebral MRI, transthoracic echocardiogram (TTE), transesophageal echocardiogram (TEE)
	Section 3.2.2 Cerebral ischemia in cor triatriatum with associated structural abnormalities	55/F/secretary	Cerebral CT-scanning, transesophageal echocardiogram (TEE)
	Section 3.2.3 Left Ventricular Non-Compaction with stroke	40/M/technician	Cerebral MRI, transthoracic echocardiogram (TTE)
Section 3.3 Stroke due to multiple etiological associations	Section 3.3 Stroke associated with patent foramen ovale (PFO), antiphospholipid syndrome, high risk thrombophilia, and dyslipidemia	46/F/teacher	Cerebral CT-scanning, angio-MRI, transesophageal echocardiogram (TEE)

**Table 2:** Summary table of the ten neurosyphilis patients diagnosed in our neurology clinic between 2010 and 2023, presenting with acute ischemic stroke.

Patient Case	Age/Sex	Presentation	Vascular Territory/Imaging	Syphilis Diagnosis	Outcome/Follow-Up
01	31/M	Acute right hemiparesis, expressive aphasia	Left MCA cortical infarct	Positive serum RPR & CSF VDRL; meningovascular neurosyphilis	Partial recovery; mild persistent aphasia and right-hand weakness after IV penicillin
02	35/F	Left arm numbness, transient facial droop	Internal capsule subcortical infarct	Reactive serum RPR; CSF pleocytosis	Good recovery; mild fine-motor deficits remain
03	42/M	Sudden right facial weakness, dysarthria	Basal ganglia infarct on MRI	CSF VDRL positive	Partial recovery; residual dysarthria and facial weakness
04	46/F	Recurrent transient ischemic attacks	Multiple cortical infarcts in MCA territory	Reactive serum & CSF VDRL	Moderate recovery; persistent mild hemiparesis and cognitive slowing
05	52/M	Hemiplegia, global aphasia	MCA + ACA infarcts; vessel wall enhancement	Meningovascular neurosyphilis confirmed by CSF	Significant residual deficits; ongoing rehabilitation needed
06	58/F	Left-sided weakness, mild confusion	Subcortical infarct; focal MCA stenosis	Serum and CSF positive	Partial recovery; persistent hemiparesis, mild cognitive impairment
07	63/M	Sudden cognitive decline, slurred speech	Cortical + subcortical multifocal infarcts	CSF VDRL reactive	Residual cognitive deficits; able to perform daily activities with assistance
08	68/F	Acute vertigo, unsteady gait, diplopia	PICA cerebellar and brainstem infarct	Serum FTA-ABS reactive; CSF VDRL positive	Partial recovery; residual mild ataxia and persistent nystagmus
09	74/M	Right homonymous hemianopsia, memory loss	Left PCA cortical infarct on MRI	Positive serum RPR (1:64); CSF pleocytosis	Stable deficits; persistent visual field defect, mild short-term memory gaps
10	85/M	Behavioral changes 4mo prior, nocturnal agitation, progressive mnesic impairment; stroke onset with global aphasia and right hemiplegia	Left sylvian artery infarct	Positive CSF TPHA; concurrent thrombocythemia	Severe right hemiplegia and global aphasia

## Data Availability

The original contributions presented in this study are included in the article. Further inquiries can be directed at the corresponding author.

## Abbreviations

The following abbreviations are used in this manuscript:

ASPECTS	Alberta Stroke Program Early CT Score
CSF	cerebrospinal fluid
ESUS	embolic stroke of unknown source
HIV	human immunodeficiency virus
MRI	magnetic resonance imagistic
NIHSS	National Institute of Health Stroke Scale
TPHA	Treponema Pallidum Hemagglutination Assay
